# How Does Chloroform Cause Death

**Published:** 1858-12

**Authors:** T. L. Maddin

**Affiliations:** Nashville


### Selections.

### HOW DOES CHLOROFORM CAUSE DEATH?

BY T. L. MADDIN, M. D., NASHVILLE.

READ BEFORE THE TENNESSEE STATE MEDICAL SOCIETY, APRIL, 1858.

[From the Transactions of the meeting of the TENNESSEE STATE MEDICAL SOCIETY, held in this city last April, we extract the following paper: it has appeared in print before, but believing it, as we do, the most profound and ingenious discussion of the phenomenon of death by Chloroform which has yet appeared, we make no apologies for including it in the present number.]

This is an inquiry which has not as yet been properly and satisfactorily analyzed. From the common occurrence of fatal results from its use, a clear and accurate knowledge of the manner by which this is brought about is of great importance to the medical practitioner, in order that he may be able to apply to the best advantages those remedies calculated to rescue his patient from imminent death. My object in this paper is briefly to institute an inquiry as to what links in the chain of life have been broken, and the best means of restoring them to their physiolcgical integrity.

It is proper that I should state here that about one hundred fatal cases from the use of chloroform have been reported. When we take into consideration the vast number of patients to whom it is administered, this mortality is relatively very small; for the universal rule is to administer it in all surgical cases of any magnitude, and it is often sportively used in operations of trivial importance. And not only so, but it is the habit of many obstetricians to administer it in their midwifery practice. Dr. Simpson, of Edinburg, uses it in every case of parturition to which he is called, and says that such is the practice of all the obstetricians of that city. In England and France it is not an uncommon practice. In the United States we find many physicians advocating its use as zealously as Dr. Simpson; among these we may class Prof. Miller, of the University of Louisville, who in a recent work on obstetrics has devoted an entire chapter to anæsthetics, and particularly chloroform.

I will state here, and I wish the fact to be specially noted, for it has an important significance in reference to the theory we will submit in this paper, that the great majority of deaths from chloroform have occurred in cases of *minor surgery*, and that not a single well authenticated fatal result has occurred in obstetrical practice. This appears rather astonishing at first view, but the result is so definite that we are forced to find its explanation in something else than mere accident, or even the philosophy of probabilities. An effect which never varies after thousands of experiments, must rest upon a cause of mathematical accuracy. Yet I do not wish to be understood to say that death can not result from the use of chloroform in parturition. The exact purport of my meaning will be better appreciated in the further development of the subject. It is proper also to state in this connection that when death has resulted in surgical cases, it is most commonly either prior or subsequent to the operation. The facts which I wish you to keep in mind, and which will find solution in the demonstration which we propose to give, are, 1st. That death has occurred from chloroform most frequently in cases of minor surgery. 2d. That it rarely occurs during the progress of an operation—occurring either before or after it. 3d. The immunity of obstetrical cases.

The manner of administering chloroform for its anæsthetic effect is by inhalation. It is received into the lungs during inspiration, and is, by the law of the diffusion of gases, mixed with the oxygen, nitrogen, and carbonic acid of the lungs. Now taking an individual, the ærial capacity of whose lungs is about a medium, in each act of respiration there are pumped in and out of the lungs about thirty to thirty-five cubic inches of air, and after an ordinary expiration there still remain in the lungs about one hundred and ten cubic inches of air, called supplemental air, but after a forcible expiration the residual quantity may still be estimated at about forty cubic inches. If we have the deepest possible inspiration to succeed this forcible expiration, a volume of about two hundred and forty cubic inches of air will be received, which added to the quantity of residual air, will give two hundred and eighty cubic inches as the ærial capacity of the lungs. It is not necessary for us to make any mention at present of the relative per cent. of oxygen, nitrogen, carbonic acid and vapor of water in the ærial mixture that is being breathed. There is no dispute in reference to the importance of oxygen to the blood, and of the elimination of carbonic acid from it, in order to preserve the integrity of physiological action, and also of the importance of a proper dilution of these active agents by nitrogen and watery vapor to the acts of expiration and inspiration. But let us apply these facts to the ordinary method of administering chloroform by inhalation. The general recommendation is to admit the free dilution of the chloroform with atmospheric air. Then suppose the respiration is quiet, and that the air breathed is about one half chloroform, after a few inspirations it is evident that one half of the supplemental air is vapor of chloroform. But it is often the case that instead of permitting the chloroform vapor to be diluted with the air, an inhaler or a napkin loaded with chloroform is placed over the nose and mouth, so as to cut off almost if not entirely the atmospheric air. Supposing still that respiration is quiet, the chloroform will soon take the place of the complemental air. When the air is thus cut off, the struggles of the patient cause forcible expiration followed by forcible inspiration; the residual air, then amounting to forty cubic inches, is immediately mixed with two hundred and forty cubic inches of chloroform vapor.

Let us upon physiological and therapeutical principles examine, in the next place, what effects will follow this condition of things. Much of this chloroform vapor by endosmose passes to the blood, and is circulated with it through different organs. What per cent. of it is requisite to saturate the blood we are unable to say. The probability is that it is not changed in its chemical condition, but circulates as a foreign body, sui generis. Its legitimate therapeutic action is to depress the encephalic centres, thus ranking it as a cerebral sedative. The portion of the cerebrum most sensitive to its impress, and which comes soonest under its influence, is the thalami optici. They being the regulators, controllers, and essential seat of sensation, when soothed into anæsthetic slumber suspend the faculty of sensation in every part of the body. It is impossible to say in what order the remaining cerebral ganglia come under its influence. But it is evident to a casual observer even, that volition, emotion, and intellection are present after sensation is suspended; remembering the fact that the corpora striata are to the motor phenomena what the thalami optici are to the sensational phenomena, and that the hemispherical ganglia, the mesocephale, and the ganglia of the base of the brain are closely linked with the corpora striata by commissures, it is readily understood how the integrity of emotional, volitional, and intellectual phenomena may remain in a measure undisturbed, and give motor expression to themselves through every part of the muscular system of animal life. If, however, the quantity of chloroform which gets access to the blood is increased, all the encephalic centres come under its anæsthetic influence, and every expression of the psychological being is suspended. We have as yet no encroachment upon the ganglia which preside over the functions of organic life, which are purely automatic, and require the integrity of the spinal ganglia, (including the medulla oblongata,) to remain in a great measure undisturbed. Many of the phenomena of animal life are simply excito-motor, the arc of action consisting in a nerve to receive and conduct a stimulus to a nervous centre or ganglia, and a nerve of motion leading from the ganglia. Most usually the excitory or sensory pole of the arc is in the integument or mucous membrane, while the motory pole is in the muscular system; the ganglia being the centre of the arc, and its integrity is essential to all the automatic phenomena both of organic and animal life. Indeed, if any link in the chain of the excito-motor arc is lost, the automatic function fails. Now if none of the organs of organic life depended upon any part of the cerebro-spinal system for their functions, then the entire system of animal life might be for a time suspended and paralyzed without involving a fatal result. But the close dependence of respiration upon the medulla oblongata links these two systems inseparably together, and if the effect of the chloroform begins to depress the spinal centres, this function is interfered with just in the same degree. If the full anæsthetic effect impresses it, then respiration necessarily ceases, for the central link in the excito-motor arc is broken. The integrity of the arc is essential to the respiratory phenomena. The medulla oblongata under the influence of chloroform, for the time involves as much the paralysis of the muscles of respiration as though this nervous centre was destroyed. Nor are they any better qualified for their work than the muscles of locomotion when the whole spinal cord is under the full sedative effect of chloroform, or is even destroyed. Now this is one of the modes by which the chloroform produces a fatal result. It will be preceded by heavy, slow, stertorous breathing. This is the only symptom in the beginning which implies that the domain of organic life is being invaded. The circulation immediately begins to fail, but this is dependent more upon the failure of the respiratory function than any direct anæsthetic effect upon the nervous centres which control the circulation. It is, however, possible to my mind that a sufficiently large quantity might be introduced into the blood to make its sedation impress synchronously upon the nervous centres of circulation and respiration.

Notwithstanding experiment, observation, and the critical examination of many reported cases of death from chloroform, sustain us in the conclusion to which we have arrived in the previous part of this paper, viz : that when the medulla oblongata is lulled into a profound anæsthetic slumber, the respiratory muscles are for the time paralyzed, the central link in the excito-motory arc is broken, and thus necessitates the suspension of the automatic respiratory phenomena ; yet we do not consider this the most fertile cause of death. There is possibly a simpler and more satisfactory explanation of this subject based upon physiological and therapeutic philosophy, to which, in all the examinations I have given the question, I have not seen the least allusion. And in order that you may comprehend the exact import of the explanation which I have to offer, you will pardon me for refreshing your minds with the physiological demonstration of the respiratory problem. The first inspiratory act of the child at its birth is brought about by the sudden change in its dynamic condition ; having been imbedded in a bag of tepid water, which was sedative to its nervous system, the sensory nerves of the entire integument, suddenly brought under the stimulus of a lower temperature, and other unaccustomed stimuli, the centers of the arc thus roused up answer to the stimulation they have received, by sending a vis nervosa along the motor nerves to the muscular system, and, in common with other muscles, the diaphragm and other inspiratory muscles are contracted, which enlarges the different diameters of the thoracic cavity, and thus, by the vacuum which is being formed, invites the air to fill the bronchial tubes, and diverts the current of blood from the ductus arteriosus into the pulmonary arteries, capillaries and veins. This is a pure excito-motory phenomenon, with the sensory pole of the arc in the general integument, the motor pole in the muscular system, and the central link in the spinal cord. But this is not the ordinary respiratory nervous arc. Each after expiration and inspiration is confined to a more specific respiratory arc. The sensory pole is changed from the general integument to the mucous membrane of the bronchial tubes and air vesicles, and consists in the distribution of the pulmonary portion of the pneumogastric nerve, the phrenic and intercostal, the motor pole, and the medulla oblongata, the central ganglia. Each act of inspiration is stimulated by the complemental or residual air, which is a mixture containing four and a third per cent. of carbonic acid. The stimulus of this mixture, and possibly that of the venous capillaries, is received by the sensory extremities of the pulmonary pneumogastric. And thus it is perceived that each respiratory act is a purely automatic phenomenon, through a special excito-motory respiratory arc. The regular respiratory phenomena are modified by volition, sensation, emotion and ideation by commissural connection between the medulla oblongata and the encephalic ganglia; and on the other hand with the excito-motory phenomena of each segment of the spinal cord, thus establishing a grand *solidarite* between the different centers of the cerebro-spinal system. The society will pardon me for this apparent digression, when they reflect that in studying the manner by which an agent produces death by suspending the function of a great organ, we must ascertain what event fails in the chain of its phenomena. It must be remembered, too, that the vapor of chloroform has a local as well as a general anæsthetic effect—if applied for a few seconds to any part of the integument, all sensibility in it is lost—it ceases to appreciate stimulation of any kind, even from rude contact, and, though it was the seat of morbid sensibility, is lulled into quiet repose. The local anæthesia is one of the most valuable of the virtues of chloroform, but it is to this effect upon the mucous membrane of the air passages, bronchial tubes and air vesicles, that its great danger is due. You will call to mind what we stated in the preceeding part of this paper in reference to the residual air, complemental air, breathing air, and ærial capacity of the lungs; and how these different airs were substituted by the vapor of chloroform, and that we arrived at the conclusion that the lungs might be filled with two hundred and eighty cubic inches of pure chloroform vapor, which by endosmosis is penetrating every part of the parenchyma of the lungs. Now the obvious consequence is that we have a local anæthesia of the sensory pole of the respiratory excito-motory arc, and as a result of this an immediate suspension of the alternate contractions of those muscles in which exist the motor pole of the same arc, and whose work it is to enlarge and diminish the thoracic cavity. The consequence is just the same as though both pneumogastric nerves had suffered section at the same instant. When there is section of both the pneumogastric nerves, the number of respirations sinks to about two or three to the minute, and the animal soon dies. The power which keeps up this imperfect respiration is due to sensational, volitional, and emotional activity. If we isolate the medulla oblongata from the nervous centers which give expression to these states of being, and thus deprive the flagging respiratory functions of their aid, section of the pneumogastrics would prove immediately fatal. This is effectually accomplished by anæthesia of the thalami optici, great centers of sensation. For if they fail to recognize the inconvenience of the partially suspended respiration, volition fails to come to the rescue. Much more does this fact hold good if all the encephalic centers are under the effects of chloroform. But if artificial respiration can be kept up for a time, this local anæsthesia will pass off, and the respiratory excito-motory arc be restored to its integrity. When we come to analyze the two explanations I have given of the manner in which chloroform produces death, we find in both cases the respiratory muscles cease to act—in the one from anæsthesia of the medulla oblongata, the central link, in the other of the sensory pole of the respiratory excito-motory arc. Now my own opinion is that the latter is the fruitful source of danger and of death, and I am further sustained in this conclusion from the fact that we have no well-authenticated case of death recorded from the anæsthetic effects of chloroform when it is swallowed into the stomach, even though very large quantities have been taken. In two cases where over an ounce had been taken in each, heavy and profound stupor supervened, attended with stertorous breathing; no chloroform was found in the contents of the stomach after using the stomach-pump, which implies that it was all absorbed; it was however detected in expiration. A case is reported in the Edinburgh Monthly Journal of Medical Science for January, 1852, where the subject was intoxicated with alcohol, and took several ounces of chloroform into his stomach at once. He died in thirty hours from a gradually increasing difficulty of respiration, attributed to the combined effects of chloroform and alcohol. A post-mortem examination discovered only heavy congestion of the lungs. This is the great pathological feature in death from chloroform inhalation, as we shall soon see. Thus we perceive that very large quantities may be circulated through the blood with great impunity when it gets access to it through the stomach, which goes far to disprove the idea that the cause of death is centric, or anæsthesia of the medulla oblongata.

We remarked in a previous part of this paper, that death rarely occurred during an operation that could be attributed purely to the chloroform, and that there was no fatal case recorded when given during parturition. Now these facts furnish us with further proof of the position we have taken. During the progress of an operation, the rough stimulation of the afferent nerves not only rouses the segment of the spinal cord of which they terminate to automatic action, but all the other segments which hold commissural connection with it, including the medulla oblongata. This *solidarite* of the spinal cord, which gives rise to the first inspiratory effort of the newborn infant, now furnishes some aid to the inspiratory efforts, when the sensory pole of the respiratory excito-motory arc is impressed with anæsthesia. The *seat of the operation* for the time becomes the *sensory pole* of this arc. This is strictly in accordance with the anatomical and physiological facts advanced in previous parts of this paper. Every one who has watched the progress of a case of labor, has observed to what a great extent the respiratory acts are influenced and modified by the pains. Indeed, we see diaphragm and other respiratory muscles controlled for the time by a different stimulation to that which ordinarily governs. The *sensory pole* is here located at the *neck of the womb* and *perineum*, and, by the commissural *solidarite* of the segments of the spinal marrow and of the medulla oblongata, in common with other segments, it controls the respiratory acts, and this will be the case even though the sensory pole of the true respiratory excito-motory arc is profoundly dormant from the effects of chloroform. Now if there was centric anæsthesia of the medula oblongta, these phenomena could not follow, and death would be inevitable in every case This substitution of the sensory pole, which is one of the conservative laws of our economy, would in this case be entirely unavailable.

It only remains for me to make a few remarks in reference to the condition of the lungs as revealed by post-mortem examinations. If the patient dies from the effects of chloroform whether the anæsthesia be at the medulla oblongata or at the sensory pole, the lungs will invariably be in a state of congestion or apoplexy; precisely such a condition as results from drowning or suffocation, or section of both pneumogastric nerves—it is a complete asphyxia. That condition which is entailed by failure of the heart, on account of failure of the respiratory phenomena, from whatever cause, is a true asphyxia. Apnœa gives rise to asphyxia. The forces of the circulation which fail first, belong to the pulmonary apparatus. 1st. The suction power exerted in consequence of an enlargement of the thoracic cavity during inspiration. 2d. The pulmonary capillary force arising out of the affinity between the blood and oxygen; the oxygen having been cut off by the manner of breathing the chloroform vapor, and also by a failure of the respiratory muscles to expand the thoracic cavity. Now the heart continuing to act, with a failure of these two forces, will necessarily entail apoplexy or capillary congestion of the lungs; a failure of the capillary force of the circulation in any organ will produce stagnation and congestion in it.

The great indication of treatment in these cases is to keep up artificial respiration until the anæsthesia shall pass off. Thus the chloroform is pumped out of the pulmonary air-passages, and oxygen supplied to the blood. If this can be instituted before the heart ceases to pulsate, a favorable result is to be confidently anticipated. The capillary stagnation begins to clear up, from a restoration of the conditions which are essential to the integrity of the two forces which were lost. The means by which this desirable result is to be accomplished, are 1st. Powerful stimulation of the integument, thus to rouse up the spinal cord, and, by the *solidarite* which links them all into unity, excite the medulla oblongata to call the respiratory muscles into action. This resource should never be neglected; immunity from danger in parturition teaches us its value; still it is not always that it can be made available, particularly when the vesicular substance of the cord is at all impressed with the sedative effect of chloroform. In connection with other stimulation, cold and warm water should be alternately dashed by the bucketful upon the surface of the body. This is a powerful stimulus to rouse up the nervous centers. The second resource to keep up respiration is the "*ready method*" of Marshall Hall, which is purely a mechanical means for expanding and contracting the thoracic cavity, and is therefore always available, and fully sufficient for any emergency, if instituted before the heart entirely ceases to beat, and all the forces of the circulation have suspended their work.—*Nashville Monthly Record.*

